# À propos d’une masse rénale droite

**DOI:** 10.11604/pamj.2020.37.37.19208

**Published:** 2020-09-09

**Authors:** Soumaya Nasri, Imane Kamaoui

**Affiliations:** 1 Service de Radiologie et d’Imagerie Médicale, CHU Mohammed VI, Oujda, Maroc

**Keywords:** Angiomyolipome, tumeur rénale, imagerie par résonance magnétique, Angiomyolipoma, renal tumor, magnetic resonance imaging

## Abstract

We report a case of a 44-year old female patient with right back pain, apyretic, without associated signs. Clinical examination showed good overall physical condition without pain in the costovertebral angle region. Lymph nodes were free. The patient underwent abdominal ultrasound and abdominal computed tomography (CT) scan which showed a lesion process measuring 09 cm along its longer axis, in the right renal lodge, roughly defined encapsulated, heterogeneous, containing calcified bulkheads. This mass was in close contact with the lower edge of the liver which was the seat of three hypodense lesions. magnetic resonance imaging (MRI) showed: spontaneously T1-hyperintense lesion (a), heterogeneous in T2 (b,c), containing few diffusion hypersignals (d). After Gadolinium injection (e) and on subtracted sequences (f) there was no fleshy component while there was peripheral contrast enhancement. The three hepatic lesions were consistent with sclerosing angiomas. Histological examination of the specimen obtained from ultrasound-guided renal biopsy showed angiomyolipoma. Angiomyolipomas are benign slow growing tumors, characterized by a proliferation of adipose tissue, vascular elements, muscle fibers in variable proportions. Abdominal CT scan is the examination of choice to make a positive diagnosis of angiomyolipoma in the vast majority of cases. Diagnosis is difficult in patients with uncommon forms, including those with no greasy component (almost pathognomonic), and with intra-tumor bleeding masking the various contingents. Differential diagnosis includes renal cancers and other benign tumors of the kidney.

## Image en médecine

Nous rapportons un cas d'une patiente âgée de 44 ans, présentant des lombalgies droites apyrétiques, sans signes associés. L'examen clinique trouve un état général conservé sans douleurs à l’ébranlement lombaire. Les aires ganglionnaires étaient libres. La patiente a bénéficié d'une échographie abdominale et d’un scanner abdominal qui ont objectivé la présence d’un processus lésionnel de 09 cm de grand axe, aux dépens de la loge rénale droite, grossièrement bien limité encapsulé de densité hétérogène renfermant des cloisons calcifiées. Cette masse présentait un contact intime avec le bord inférieur du foie qui était le siège de 03 lésions hypodenses. A l'imagerie par résonance magnétique (IRM): ce processus était spontanément hyper intense en T1 (a), hétérogène en T2 (b,c), renfermant quelques hypersignaux à la séquence de diffusion (d). Après injection de Gadolinium (e) et sur les séquences de soustraction (f). Il n'existe pas de composante charnue et on note un produit de contraste (PDC) périphérique. Les trois lésions hépatiques étaient compatibles avec des angiomes scléreux. Une biopsie rénale échoguidée pour une étude histologique était en faveur d'un angiomyolipome (AML). Les angiomyolipomes sont des tumeurs bénignes, d'évolution lente, caractérisées par une prolifération faite de tissu adipeux, d'éléments vasculaires, de fibres musculaires à des proportions variables. La tomodensitométrie abdominale est l'examen de choix permettant de poser le diagnostic positif d'AML dans la grande majorité des cas. Le diagnostic est difficile dans les formes atypiques: notamment en absence de la composante graisseuse quasi pathognomonique et s’il y a une hémorragie intra-tumorale importante masquant les différents contingents. Le diagnostic différentiel se fait alors avec les tumeurs rénales malignes; les autres tumeurs bénignes du rein.

**Figure 1:**
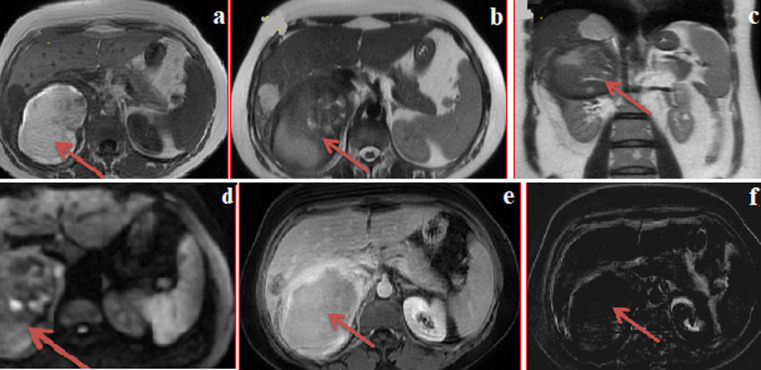
angiomyolipome rénal droit; a) séquence T1; b) séquence T2 axiale; c) séquence T2 coronale; d) séquence de diffusion; e) séquence T1 injectée; f) séquence de soustraction

